# 

**Published:** 1903-10-10

**Authors:** 


					JUST ISSUED. EIGHTH EDITION. Grown 8vo, 10s. 6d.
PHARMACY, MATERIA MED1CA
AND THERAPEUTICS
Ii now rewritten and printed in larpa type, and ia made a companion to
Ihe following volume. It ontains tn account of the action of every
drug in the B.P., aa well as a acction upon all the newest drugs, with
*b Introduction to the Art o f Dispensing, With woodcut i, prescriptions, & a.
Also FOURTH EDITION. 1055 page*, 8vo. 10s.
DICTIONARY OF TREATMENT.
Containing a Revised and up-to-date Article upon every Diseased Con-
dition, whether Medical, Surgical, Gynaecological, or Ophthalmologics^
aa well as Articles upon Poisoning, Drowning, and the various Surgical
Emergencies and Accidents, arranged for Rapid Reference
By W. WHITLA., M.A., M.D., Professor of Materia Medics, Qmeeu'i
College, Belfast; Senior Physician to the Royal Victoria Hospital, &o.
The Publisher will supply botb volumes, carriage paid, for 21/-
T.nnrtn" : RKTJRV RKMSTTAW. Sfifi Strap*.
THE HANDBOOK OF THE OPEN-AIR TREATMENT
By Dr. Charles Reinhardt
(Visiting Physician to Hailey Sanatorium, Wallingford, and Stourfleld Park
Sanatorium, Bournemouth),
Contains Views, Descriptions, and Information respecting
30 Sanatoria in Great Britain.
Price: Cloth, 2/6; Paper, 1/- (postage 4d.)
The " HEALTH RESORT " Publishing Office, 379 Strand, London, W.O.
WORKS BY DAVID CTAIjSH, H.D.
ROMTGEN RAYS IN MEDIOAL WORK. Third Edition. 111. Bd. Bet.
Baillifere & Oox, London.
THE HAIR AND IT8 DISEASES. 8e.6d.net. Bailii&re & Oox.
GOLDEN RULES OF 8KIN PRAOTIOE. ls.net. Wright A Co., Brbtol.
*05 AMO OLD AGE : a Handbook. 2s. Rd. R. A. Kverett <Sc Oo.. L<tnd??
THE BRISTOL MEDICO-CHIRURGIGAL
JOURNAL.
Published under the auspices of the Bristol Medico?
Chirurgical Society.
Editor : R. SHINGLETON SMITH, M D., B.Sc.
with whom are associated
J. Michhll Clabke, M.A., M.D.,
J. Lacy Firth, M.S., M.D., James Swain, M.S., M.D.
P. Watson Williams, M.D., Assistant Editor.
Editorial Secretary : James Taylor.
CONTENTS OF SEPTEMBER NUMBER.
Joseph Griffiths Swayne, M.D
(Lond).
Bound the World. By R. Rox-
burgh, M.B., F.R.C.S.Ed.
A Contribution to the Con-
servative Surgery of the
Uterine Appendages. By
James Swain, il.S., iil.D.Lond,
F.fi.O.S.
A Case of Perforated Duodenal
Ulcer, with remarks. By
G. Munro smith.
The Antibodies in Disease. By
E. Bushnell, M.D.Lond.
A Granuloma of the Nose, Due
to Iodide of Potassium. By
0 Percival Orouch, M.B.Lond.,
F.R.O.S.
The Practical Value of Blood-
Counts. By William Gordon,
M.A., M.D.Oantab.
Four Cases of Benal Calculi
Treated Successfully by
Lumbar Nephro-Lithotomy.
By ii. J. Trevor Jones, M.JJ.
Brux-., M.R.O.S., l.R.C.P.Lond.
Progress of the i Medical
sciences:
Medicine. G. Parker, M.D.,
M.A.Cantab
Surgery. T. Oarwardine, M.S.,
M.B.Lond.. F.R.0 S.
E1 e e t r o-T herapeutics.
J. Taylor.
Beviews of Books.
Editorial Notes.
Notes on Preparations for the
Sick.
The Library of the Bristol
Medico-Chirurgical Society.
Obituary Notices.
Local Medical Notes.
Price One Shilling and Sixpence.
BRISTOL: J. W. ARROWSMITH.
London : J. & A. Churchill, 7 Great Marlborough Street.
28 THE HOSPITAL. Oct. 10, 1903.
NOTICE TO CORRESPONDENTS.
All MSS., Letters, Books for Review, and other matters intended for the
?diior should be addressed Thb Editor, " The Hospital,"_The Hospital
Buildings, 28 & 29 Southampton Street, Strand, W.O.
Tis Editor cannot undertake to return rejected MS., even when
Hasompsnied by stamped directed envelope.
CTotlce-'.ta Advertisers and' Subscribers.
?.11 Advertisements,- Order3 for CopiesJof The Hospital, and Business
Communications should be addressed to THE XflATJilGER,
('?ZTot to the Editor), The Hospital,|28 & 29 Southampton Street,
Strand, London, W.O.
The Cost of Subscription, post free, is as follows.?Per week, 3jd.; per
qnartar, 3s. 9d.; per half-year, 73. 6d.; per annum, 15s. Foreign and
ColonialPer annum, 19s. 6d., payable, in all cases, in^advance.
HOTICE.?Vol. XZ3SXXI. of "The Hospital" (October
1502 to March 1903) In handsome cloth binding:,
will shortly belon sale at the office of this Journal,
price 7s. 6d. Cases for binding- the Numbers
contained In this Volume Is. 6d. Index to Vol.
2?2?XXXX., 2}d., post free.
Sho zaonthly part for October Is now ready.
Price Is., by post Is. 3d.
BOOKS RECEIVED.
Messes. Kegan Paul, Trench, Trubner and Co.
Home Nursing." By J. Moffett.
J. "Wright and Co., Bristol.
" Golden Rules for Diseases of Children." By George
Carpenter.
A. II. Jones.
" Rearing of the Infant." By II. G. Ivellett.
The Student of Medicine,
APPOIJfTMBKTS.
John Brown, M.D., D.P.IT.Vict.., Univ. Manchester, reap-
pointed Medical Officer of Health for the Borough of Bacup, also
Physician to the Southall Isolation Hospital. E. C. Garland,
L.R.C.P.Edin., M.R.C.S.Eng., reappointed Medical Officer of
Health for the Urban District of Yeovil. Francis Alex.
Hardy, M.D., C.M., Medical Officer and Public Vaccinator to the
Fulbeck District of the Newark Union and to the Leadenham
District of the Sleaford Union. E. H. Harrison, M.B., Certi-
fying Factory Surgeon for the St. Neots District, Huntingdonshire.
W. L. A. Leslie, M.B.Aberd., Assistant Medical Officer to the
Grahamstown Asylum, South Africa. H. It. Langmore, M.B.,
B.C.Cantab., District Medical Officer of the Wallingford Union.
It. It. Law, M.D., B.C., Clinical Assistant to the Chelsea Hospital
for Women. E. J. Hyan, M.D.McGill, L.R.C.P.&S.Edin., Dis-
trict Medical Officer of the Easington Union. Eaymond Henry
Shaw, M.S., M.B.Dunelm, Assistant Surgeon to the Great Yar-
mouth Hospital. E. H. Snell, M.D.. B.Sc.Lond., D.P.II. Camb.,
F.R.S.E., permanently reappointed Medical Officer of Health to
the City of Coventry. W. Thomas, L.R.C.P.&S.Edin., District
Medical Officer of the Anglesey Union. W. E. Thomas, M.D.,
Medical Officer of the Workhouse of the Pontypridd Union.
G. J. K. Turner, M.B., C.M.Aberd., District Medical Officer of
the Dulverton Union. Charles Edward Wallace, M.R.C.S.,
L.R.C.P., L.D.S., Dental Surgeon to Feltham School under the
London County Council. Lewis Worts, L.R.C.P.&S.Edin.,
Assistant Medical Superintendent and Dispenser at the Croydon
Infirmary. C. Y. Young, M.D., M.Ch., Clinical Assistant to the
Chelsea Hospital for Women.
? The Hospital" Institutional ll>lbrary.
" Hospitals and Asylums of the World." 4 Vols., with a Port-
folio of Plans, complete ?12 12s.
" Burdett's Hospitals and Charities, 1903." 5s. net.
" Burdett's Official Nursing Directory, 1903." 3s. net.
" Cottage Hospitals, General, Fever, and Convalescent." 10s. 6d.
" Uniform System of Accounts for Hospitals and Public Institu-
tions." New Edition. 4s. net.
" Hospital Expenditure: The Commissariat." 2s. 6d.
"A Legal Handbook for the Use of Hospital Authorities."
2s. 6d.
"The Cottage Hospital Case Book or Register of Patients." 10s.
and 12s. 6d.
All these are published by the Scientific Press, Ltd., and may
be obtained through any bookseller, or direct from the publishers,
28 and 29 Southampton Street, Strand. London, W.C.
24 Nursing Section. THE HOSPITAL. Oct; 10, 1903.
STANDARD NURSING MANUALS.
A HANDBOOK FOR NURSES.
By J. K. Watson, M.D., M.B., O.M.
Second Edition. Crown 8vo. ow 400 pp, profusely illustrated,
5s. net, 5s. 5d. post free.
" A concise and comprehensive exposition of as much
medical knowledge as a nurse needs. The work cannot but
prove useful to those for whom it has been designed."
Scotsman.
NURSING: ITS THEORY AND PRACTICE.
By Percy G. Lewis, M.D., H.B.O.S., L.S.A.
New and Enlarged Edition.
drawn 8vo. over 400 pp. profusely illustrated, 3s. 6d. post free.
"Nurses will find the book full of interest and instruc-
tion, which cannot fail to assist them in their work."
British Medical Journal.
NURSING: ITS PRINCIPLES AND
PRACTICE.
By Isabel Adams Hampton.
Kew and Enlarged Edition.
Socoud Edition. Grown 8^0. illustrated, 7s. 6d. net,
post free 7s. lOd.
"The subject has been scientifically, carefully, and com-
pletely treated by Miss Hampton."?The Hospital.
OPHTHALMIC NURSING.
By Sydney Stephenson, M.B., F.B.O.S.E.
Second Edition. Crown 8vo. illustrated, with glossary of
terms, cloth, 3s. 6d. net, 3s. lOd. post free.
" May very advantageously be studied by nurses who
ace about to enter the Ophthalmic service of a hospital."
Lancet.
NURSING IN DISEASES OF THE THROAT,
NOSE AND EAR.
By P. Macleod Yearsley, F.R.O.S.Eng., M.B.O.S.,
L.B.O.P.Lond.
Bemy 8vo. Illustrated, cloth, 2s. 6d. post free.
"Of practical service to every nurse who wishes to be
thoroughly trained."
Scottish Medical and Surgical Journal.
SURGICAL WARD-WORK AND NURSING.
By Alexander Miles, M.D.Edin., O.M., F.B.O.S.E.
He vised Edition. Demy 8vo. Illustrated, cloth gilt, 3s. 6d.
net, 3s. lOd. post free.
" An illustrated manual which will furnish much needed
guidance to the student and the nurse in the early stages of
tbeir apprenticeship."?The Times.
ELEMENTARY ANATOMY AND SURGERY
FOR NURSES.
By William McAdam Eccles, M.S.Lond., M.B., &c.
Crown 8vo. Illustrated, cloth, 2s. 6d. post free.
" Valuable to nurses who are beginning the study of
anatomy. The instruction is given very clearly and con-
cisely, and the reader is able to glean much information in
a very condensed form."?Nursing Notes.
ELEMENTARY PHYSIOLOGY FOR
NURSES.
By O. F. Marshall, M.D., B.Sc., F.R.O.S.
Crown 8vo. Illustrated, cloth, 2s. post free.
"Nurses will find in it all that they require to know of
the subjects treated."?Daily Chronicle.
PRACTICAL GUIDE TO SURGICAL
BANDAGING. AND DRESSINGS.
By Wm. Johnson Smith, F.It.C.S.
Demy lGmo., profusely Illustrated (suitable for apron
pocket), cloth, 2s. post free.
ART OF MASSAGE.
By A. Creighton Hale.
Second Edition. Demy 8vo. Profusely illustrated, cloth gilt,
6s. post free.
" Mrs. Hale seems to have had considerable experience,
not only as a practitioner, but as a teacher of the art, and
explains in a series of clearly illustrated articles the many
complaints for which massage has been found beneficial,
and the special manipulations required in each case."
Morning Post.
ART OF FEEDING THE INVALID.
By a Medical Practitioner and a Lady Professor of Cookery.
New and Popular Edition. Demy 8vo. cloth,
Is. 6d. post free.
" A new edition of this valuable work is very welcome,
and we heartily commend it to all who desire reliable and
practical instruction in sick cookery."?Nursing Notes.
COMPLETE CATALOGUE POST FREE ON APPLICATION.
HOSPITAL SISTERS AND THEIR DUTIES.
By Eva C. E. Lucres, Matron to the London Hospital
Third Edition. Crown 8vo. Cloth gilt, 2s. 6d. post free.
" An admirable guide to the beginner anxious to take up
a Sister's work. Its pages bring help and knowledge im-
parted in a very sympathetic, straightforward manner by
one who has had practical experience in the lesson she
teaches."?Gentleicoman.
The SCIENTIFIC PBESS, LTD., 28 & 29 Southampton Street, Strand, London, W.C.
xxiv Nursing Section. THE HOSPITAL. Oct. 10, 1903.
Standard Works \ CHURCHILL S Latd M[m
Books for* Nurses.
NURSING.
Nursing, General, Medical and Sur-
gical, with an Appendix on Sick
Room Cookery. By Wilfred J. Hadlby,
M.D., F.R.O.P., F.R.O.S., Physician and
Pathologist to the London Hospital, Lec-
turer to Nurses at tha London Hospital
Nursing School .... 3s. 6d.
MEDICINE.
Leetures on Medicine to Nurses. By
Herbert E. Cutf, M.D., P.R.O.S., Medical
Superintendent, North Eastern Fever Hos-
pital, London. Fourth Edition. With 29
Illustrations . . . . ? 3s. 6d.
INFANT FEEDING.
The Natural and Artificial Methods of
Feeding Infants and Young Children.
By Edmund Oautley, H.D.. F.RC.P.,
Physician to the Belgrave Hospital for
Children; late Casualty Physician and
Assistant Demonstrator of Physiology, St.
Bartholomew's Hospital. Second Edition.
7s. 6d.
DIET.
Diet and Food considered in relation
to Strength and Power of En-
durance, Training and Athletics.
By A. Haig, M.D., F.R.O.P. Fourth Edition.
2s.
MIDWIFERY.
A Short Practice of Midwifery for
Nurses. By Henry Jellett, M.D. (Dub
Univ.), F.R.C.P.I., Ex-Assistant Master,
Rotunda ^Hospital, Dublin, Extra Examiner
In Midwifery and Gynecology, Royal College
of Physicians of Ireland, Extern Examiner
in Midwifery, Royal University of Ireland.
With 67 Illustrations .... 6s.
MONTHLY NURSING.
A Short Manual for Monthly Nurses.
By 0. J. Culling worth, M.D., F.R.C.P.,
Obstetric Phyeician to St. Thomas's Hos-
pital. Revised with the assistance of M. A.
Atkinson, Matron of the General Lying-in
Hospital, London. Fifth Edition. Is. 6d.
HEALTH OF A WIFE.
Chavasse's Advice to a Wife on the
Management of her Own Health.
Fourteenth Edition. By Fancourt Barnes,
M.D., Consulting Physician to the British
Lying-in Hospital .... 2s. 6d.
HEALTH OF CHILDREN.
Chavasse's Adviee to a Mother on
the Management of her Children.
Fifteenth Edition. By George Carpenter,
M.D., Physician to the Evelina Hospital for
Children 2s. 6d.
ANTISEPTICS.
Lessons in Disinfection and Sterilisa-
tion ; an Elementary Course of Bacterio-
logy, with a Scheme of Practical Experi-
ments. By F. W. Andrewes, M.D., F.R.C.P.,
Lecturer on Pathology at St. Bartholomew's
Hospital. With Illustrations . 3s. net.
\Just Published,
SURGERY.
A Manual of Minor Surgery and
Bandaging. By Christopher Heath,
F.R.O.S., and Bilton Pollard, F.R.O.S.,
Surgeon to University College Hospital.
Twelfth Edition. With 195 Illustrations.
6s. 6d.
HYGIENE.
The Elements of Health: An Introduc-
tion to the Study of Hygiene. By Louis C.
Parkes, M.D., D.P.H.Lond., Lecturer on
Public Health at St. George's Hospital.
3s. 6d.
DICTIONARY.
A Short Dictionary of Medical Terms.
By R. G. Maynk. M D.. LL.D. . 2s. 6d.
7 GREAT MARLBOROUGH STREET, LONDON.

				

